# Perfluorosulfonic Acid Membranes Thermally Treated and Modified by Dopants with Proton-Acceptor Properties for Asparaginate and Potassium Ions Determination in Pharmaceuticals

**DOI:** 10.3390/membranes9110142

**Published:** 2019-10-30

**Authors:** Anna Parshina, Tatyana Kolganova, Ekaterina Safronova, Alexander Osipov, Ekaterina Lapshina, Anastasia Yelnikova, Olga Bobreshova, Andrey Yaroslavtsev

**Affiliations:** 1 Department of Analytical Chemistry, Voronezh State University, Universitetskaya pl., 1, Voronezh 394018, Russia; tanyadenisova@list.ru (T.K.); ekaterina.l.u@mail.ru (E.L.); bobreshova@chem.vsu.ru (O.B.); 2 Kurnakov Institute of General and Inorganic Chemistry RAS, Leninsky pr., 31, Moscow 119991, Russia; ekvoropaeva@yandex.ru (E.S.); osipov.aleksander.k@gmail.com (A.O.); yaroslav@igic.ras.ru (A.Y.); 3 Department of Chemistry, Moscow State University, Leninskie Gory, 1(3), Moscow 119991, Russia

**Keywords:** potentiometric multisensory systems, cross sensitivity, DP-sensors, perfluorosulfonic acid cation exchange membranes, silica, membrane thermal treatment, membrane mechanical deformation, aspartic acid, pharmaceuticals

## Abstract

The influence of incorporation of the dopants with proton-acceptor properties into perfluorosulfonic acid cation exchange membranes (MF-4SC and Nafion), and their treatment conditions on the characteristics of Donnan potential (DP)-sensors (analytical signal is the Donnan potential) in the aqueous solutions containing asparaginate and potassium ions in a wide pH range was investigated. A silica, surface modified by 3-aminopropyl and 3-(2-imidazolin-1-yl)-propyl groups, was used as the dopant. The membranes were subjected to mechanical deformation and thermal treatment at various relative humidities. The relationship between water uptake and diffusion permeability of membranes subjected to modification and treatment and the cross sensitivity of DP-sensors based on them to counter and co-ions was studied. The multisensory systems for the simultaneous determination of asparaginate and potassium ions in a concentration range from 1.0 × 10^−4^ to 1.0 × 10^−2^ M and pH range from 4 to 8 were developed. An array of cross-sensitive DP-sensors based on MF-4SC membranes containing 3 wt.% SiO_2_ modified by 10 mol.% 3-aminopropyl and 3-(2-imidazolin-1-yl)-propyl was used for the potassium asparaginate hemihydrate and magnesium asparaginate pentahydrate determination in Panangin^®^ (with an error of 2 and 4%, respectively).

## 1. Introduction

Asparaginates of alkali, alkaline earth, and some transition metals are active substances of antiarrhythmic pharmaceuticals. Voltammetric sensors are used for determination of asparaginate ions in pharmaceutical and physiological environments most often [1,2,3]. Amperometric [4] and fluorimetric [5] sensors (Table 1) are known for these purposes also. Sensors based on graphite electrodes modified by a molecularly imprinted polymer film with titanium dioxide nanoparticles and multiwall carbon nanotubes (MWCNTs) [1] or by gold nanoparticles and MWCNTs [2] were developed for determination of D- and L-aspartic acid in the model solutions, blood serum, cerebrospinal fluid, and pharmaceuticals using differential pulse inversion voltammetry. Carbon paste electrodes modified by copper micro- and nanoparticles were proposed for the investigation of electrocatalytic oxidation and for the determination of amino acids, in particular, aspartic acid, in the model solutions [3]. An amperometric biosensor based on platinum electrode with an enzyme membrane immobilized on its surface was designed for determination of L-aspartic acid in the model solutions [4].

In most cases, such sensors are characterized by low detection limits and the high accuracy of analyte determination. The short lifetime of an active layer, as well as the special requirements for the experiment conditions due to the need of the high diluted analytes to eliminate the influence of matrix effects and to prevent of layer contamination could be mentioned among their disadvantages (Table 1). Potentiometric sensors are promising for analysis of pharmaceuticals with a relatively high content of active substances.

The polyionic composition of pharmaceuticals, as well as the use of alkaline hydrolysis in the preparation of aspartic acid, define the relevance of aspartic ion determination simultaneously with alkali metal cations in a wide pH range. The application of the multisensory approach [6] allows to take into account the influence of several analytes on the response of cross-sensitive sensors simultaneously. A voltammetric multisensory system based on glassy carbon electrodes modified by polyarylenephthalides was developed for the identification of antiarrhythmic drugs included in the β-blockers group [7]. The arrays of potentiometric sensors with plasticized polymer membranes based on organic tetraalkylammonium ion exchangers with penicillin antibiotic anions were proposed for the separate determination of β-lactam antibiotics in the model mixtures and pharmaceuticals [8]. A system based on eight miniaturized potentiometric sensors was used for determination of acetaminophen, ascorbic acid, and acetylsalicylic acid in the presence of various caffeine amounts under flow injection analysis conditions [9].

The application of hybrid materials in electrochemical sensors [10,11,12] and multisensory systems [13,14,15,16] provides additional opportunities for variation of their characteristics [17]. The use of polymer films (perfluorosulfonic acid cation exchange Nafion-type membranes, polypyrrole, polythiophene, polyaniline, etc.) containing inorganic nanomaterials or molecular dopants solves the problem of low sensitive layer adhesion to the electrode surface and materials serve as ion-electron converters in potentiometric solid state sensors [18]. The use of Nafion-type membranes in such sensors prevents the sorption of redox active compounds poisoning the electrode material and increases the analyte sorption. The unique properties of perfluorosulfonic acid cation exchange membranes are caused by their structural features: the separation of hydrophilic and hydrophobic areas leads to the formation of pores and channels that can selectively sorb and transfer ions [19]. The cross sensitive potentiometric sensors based on modified Nafion-type membranes with gradient dopant distribution were proposed in [20,21]. The sensors have special design: the membrane is fixed in the sensor’s body so that the distance between the internal reference solution and the test solution corresponds to the membrane length. The analytical signal of such sensors is the Donnan potential (DP) at the boundary between the modified part of membrane and the test solution. It was shown that the use of Nafion-type membranes with a silica nanoparticles surface modified by proton-acceptor groups allows to vary the sensitivity of DP-sensors to anions and zwitterions of aspartic and glutamic acids incoming into the membrane by non-exchange sorption [22]. This is due to the presence of two types of sorption centers (acidic and basic) in the membrane, as well as due to the influence of hydration and the volume of membrane pores on the concentration and conformation of analyte ions. It is known that a change in the hydration of Nafion-type membranes, as well as the conformational transformations of polymer chains influencing on the distribution of sulfo groups inside the material and on the pore size, can be achieved by thermal treatment at the various relative humidities and the mechanical deformation of them [23,24,25,26,27,28]. Such changes are difficult at room temperature, i.e., are irreversible (“memory effect” of membranes). The possibility for a significant increase in DP-sensor sensitivity to methionine amino acid anions and zwitterions by the use of membranes treated under hydrothermal conditions or subjected to deformation with a subsequent thermal treatment was shown in [29]. The obtained results allow to suppose that the same treatment of unmodified and hybrid membranes will give an opportunity to vary the number and availability of sorption centers for asparaginate ions.

The aim of this work was the development of a multisensory system with DP-sensors based on perfluorosulfonic acid cation exchange membranes (MF-4SC and Nafion) modified by silica with nitrogen-containing groups, as well as the investigation of thermal treatment at the various relative humidities and mechanical deformation influence on the simultaneous determination of asparaginate and potassium ions in the aqueous solutions and pharmaceuticals.

## 2. Materials and Methods

### 2.1. Materials and Reagents Used

MF-4SC (Plastpolymer, St. Petersburg, Russia, dry membrane thickness ~140 μm, ion-exchange capacity (IEC) was 1 mmol/g, equivalent weight was 1200) and Nafion 115 (Aldrich, St. Louis, MO, USA, dry membrane thickness was 130–140 μm, IEC ~0.95 mmol/g, equivalent weight was 1100) membranes obtained by extrusion; a solution of perfluorosulfonic acid polymer in the H^+^-form in isopropyl alcohol (MF-4SC, Plastpolymer, St. Petersburg, Russia, concentration was 10.0 wt.%, IEC was ~0.95 mmol/g, equivalent weight was 1100); 3-aminopropyltrimethoxysilane (Fluka, 98%, Buchs, Switzerland); 3-(2-imidazolin-1-yl)propyltriethoxysilane (Fluka, 98%, Buchs, Switzerland); aqueous ammonia (Himmed, >99%, Moscow, Russia); hydrochloric acid (Himmed, >99%, Moscow, Russia); sodium chloride (Himmed, >99%, Moscow, Russia); potassium hydroxide (Ecohim, standard-titer, Moscow, Russia); aspartic acid (2-aminobutanedioic acid, Sigma-Aldrich, >99%, St. Louis, MO, USA); Panangin^®^ (Gedeon Richter, Budapest, Hungary, concentrate for preparation of infusion solution); deionized water (resistance was 18.2 MΩ).

### 2.2. Membrane Preparation

Hybrid materials for DP-sensors were obtained so that only half of the membrane length contained dopant. This part of the film was in contact with the test solution when the sensor response was determined. The membrane part contacting with the reference solution of DP-sensor was not modified. Uniformly modified membranes samples were obtained to study the IEC, water uptake, and diffusion permeability.

#### 2.2.1. Preparation of Hybrid Membranes by Casting

MF-4SC membranes containing 3 wt.% SiO_2_ with 3-aminopropyl-(R1) and 3-(2-imidazolin-1-yl) propyl-(R2) groups (with concentrations equal to 5 and 10 mol.% from the oxide amount) on the surface were obtained by casting according to the procedure described in [30]. The polymer solutions in the presence of a calculated precursor amounts (tetraethoxysilane and 3-aminopropyltrimethoxysilane or 3-(2-imidazolin-1-yl) propyltriethoxysilane) were homogenized by stirring on a magnetic stirrer (1400 rpm) for 1 h, then placed on a Petri dish and dried in air at room temperature during 24 h, then kept at 60 °C (for 1 h), 70 °C (for 1 h), 80 °C (for 1 h), 85 °C (for 1 h), 60 °C (for 4 h) for solvent evaporation and the film formation. The films were removed from the glass surface and pressed at 110 °C under pressure of 5 MPa for the improvement of mechanical properties. Hydrolysis of precursors was carried out at the final stage by membrane treatment with 10% ammonia solution during 30 min under stirring to form dopants. The obtained materials were conditioned by treatment in 5% hydrochloric acid solution for 3 h at the room temperature followed by multiple washing in deionized water (the cycle was performed twice). Using NMR spectroscopy (31P Bruker MSL-300 spectrometer, Bruker, Karlsruhe, Germany), it was shown that nitrogen-containing groups in silica samples obtained by a similar method outside the membrane matrix are localized on the nanoparticle surface [31].

#### 2.2.2. Preparation of Hybrid Membranes In Situ

Nafion membranes containing SiO_2_ modified by R1 and R2 were prepared in situ. The Nafion 117 extrusion membranes were first treated by alcohol solutions of precursors mixed in a given ratio (10 mol.% of 3-aminopropyltrimethoxysilane or 3-(2-imidazolin-1-yl)propyltriethoxysilane from the tetraethoxysilane amount) for 3 h at room temperature under continuous stirring. After this, the membranes were treated with a 10% aqueous ammonia solution at the room temperature under continuous stirring for 30 min to form dopant particles in their matrix. The obtained materials were conditioned by treatment in a 5% hydrochloric acid solution at room temperature for 3 h followed by multiple washing in deionized water (the cycle was performed twice).

#### 2.2.3. Membrane Treatment

The conditions of membrane treatment were selected based on previously obtained data on the changes of the water uptake, conductivity, permeability, and selectivity of MF-4SC membranes as a result of thermal, hydrothermal treatment, and mechanical deformation [25]. The membranes were conditioned and transferred to the K^+^-form before the thermal treatment and deformation. Therefore, the membranes were kept in 5% HCl solution at room temperature for 3 h, washed in the deionized water until disappearance of reaction to chloride ions (the cycle was carried out twice), then treated by 2 M KCl solution for 72 h and washed in deionized water again. The membranes were kept in deionized water at room temperature at least for 72 h after treatment or deformation and were stored in the deionized water. The thermal treatment of unmodified and hybrid membranes was carried out in a hydrated state at a relative humidity (RH) of 60% and temperature t = 95 °C and in the contact with water (t_ht_ = 120 °C, ht—hydrothermal treatment). The Binder MKF115 climate chamber (Binder, Tuttlingen, Germany) was used to set the required humidity and temperature. Pre-dried unmodified MF-4SC and Nafion samples were subjected to mechanical deformation. The deformation was carried out by uniaxial tension up to 80% from the initial length using a Tinius Olsen H5KT universal tensile testing machine (Tinius Olsen, Salfords, UK) with a 100 N force sensor at the temperature of t = 27 ± 2 °C and at the relative humidity of RH = 20 ± 2% with a deformation rate of 5 mm/min. Then the membranes were kept in vacuum at 80 °C for 12 h and conditioned in 5% hydrochloric acid solution at room temperature for 3 h followed by multiple washing in deionized water for stabilization.

### 2.3. Membrane Regeneration

The membrane regeneration after use in DP-sensors was performed by means of their transfer into the initial K^+^-form. The membranes were kept in 0.1 M KCl solution for 30 min under continuous stirring and were stored in deionized water between the series of repeated measurements (~100 measurements). Membranes were placed in deionized water after each measurement. The membranes were equilibrated with 2 M KCl solution for 72 h, and then washed in deionized water after prolonged use (up to 3 months).

### 2.4. Apparatus and Procedures

The concentration of dopant in membranes was determined by thermogravimetric analysis taking into account the mass of residue after samples annealing in a dry state at 600 °C. The particle size of incorporated nanoparticles was determined by transmission electron microscopy (TEM) on a JEOL JEM-2100 equipment (accelerating voltage of 200 kV, JEOL, Tokyo, Japan). Prior to the experiment the hybrid membrane samples were dispersed by sonication in methanol. The chemical composition of hybrid membranes was analyzed using a Carl Zeiss NVision 40 scanning electron microscope (accelerating voltage of 1 kV, Carl Zeiss, Oberkochen, Germany) with an energy-dispersive X-ray (EDX) analysis attachment. The acquisition time of the EDX analysis was 40 s and each sample was investigated at least in five spots.

The membrane’s IEC was determined by titration using an Expert-001 pH meter (Econix-Expert, Moscow, Russia). A portion of the air-dry membrane in the H^+^-form was kept in 0.5 M NaCl solution for 24 h with continuous stirring. Then the solution was titrated by 0.01 M NaOH solution.

The thermal analysis of membranes was carried out using Netzsch-TG 209 F1 thermobalance (Netzsch, Selb, Germany) in an argon atmosphere in the platinum crucibles in the temperature range from 25 to 200 °C (heating rate was 10 deg/min). The membranes stored in deionized water were taken out and wiped with filter paper immediately prior to measurements. The water uptake (*ω* (H_2_O), %) was calculated by the formula:

(1) $$\omega (H_{2}O)=\frac{\Delta m}{m}\cdot 100$$

where Δ*m* is the difference between the samples mass before the thermal treatment and after 200 °C (g), *m* is the sample mass before the thermal treatment (g).

Determination of the diffusion permeability was carried out as follows. The membrane was placed in a cell between two chambers, the volume of each of which was 32 cm^3^. The electrolyte solution (0.1 M KCl or HCl) was placed into a chamber on one of the membrane sides and the deionized water was placed into the other chamber. The change of the pH value and electrical conductivity of solution was measured during the experiment using an Expert-001 pH meter (Econix-Expert, Moscow, Russia) or an Expert-002 conductometer (Econix-Expert, Moscow, Russia) in a chamber with deionized water. The diffusion permeability of membranes was calculated by the formula:

(2) $$P=\frac{dc}{dt}\cdot \frac{V\cdot l}{S\cdot \Delta c}$$

where *V* is the solution volume (32 cm^3^); *l* is the membrane thickness (cm); Δ*c* is the concentration gradient (mol/cm^3^); *t* is the time (s); *S* is the active membrane area (4.9 cm^2^). The error of *P* determination is less than 1%.

**Figure 1:**
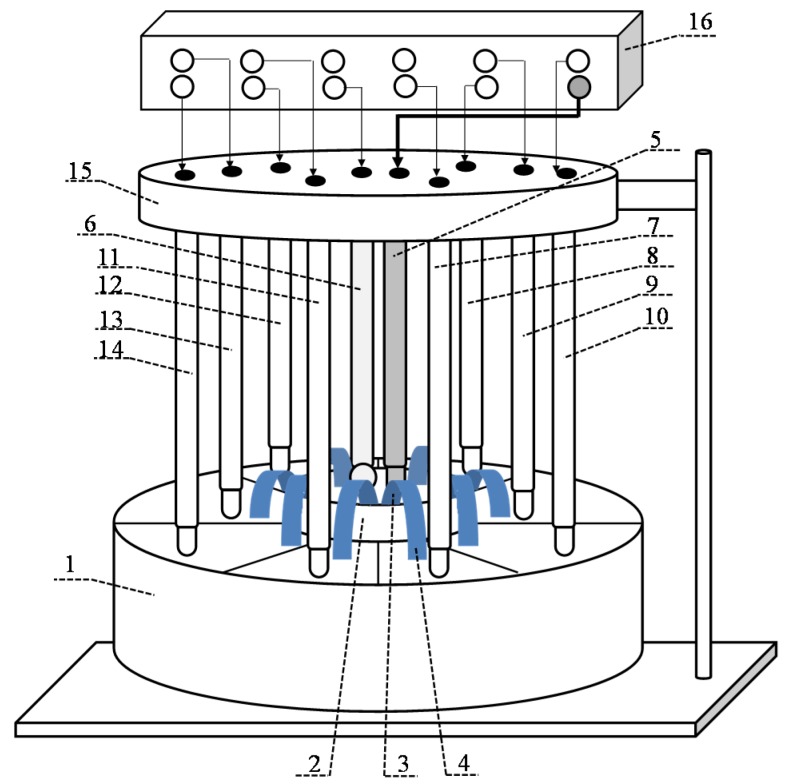
A cell for evaluation of the Donnan potential (DP)-sensor system responses: 1—shell for the reference solution; 2—shell for the test solution; 3, 4—modified and unmodified membrane ends in contact with the test solution and the reference solution, respectively; 5—silver chloride electrode immersed in the test solution; 6—glass electrode for pH measurement; 7–14—silver chloride electrodes immersed in a reference solution; 15—holder for electrodes; 16—multi-channel potentiometer [22].

The cell for evaluation of responses of DP-sensors system included two shells based on non-conducting material, a set of membranes with various composition, silver chloride electrodes, and a multichannel potentiometer (Figure 1) [22]. The inner shell (d = 4.5 cm, h = 3.5 cm) was filled by the test solution. The outer shell included one section (d = 5 cm, h = 3 cm) for the inner shell and the eight sections (V = 28 cm^3^) for the reference solution (1 M KCl). One end of the membrane was immersed into the test solution and another end was immersed into the sections with the reference solution. When using hybrid membranes, the modified end was immersed into the test solution. A silver chloride electrode (RE-10103, Econix-Expert, Moscow, Russia) connected to the input of a multi-channel potentiometer for a reference electrode was immersed into the test solution, and the silver chloride electrodes (RE-10103, Sensor Systems, St. Petersburg, Russia) connected to the measurement inputs were immersed into the section with the reference solution. The voltage of several circuits (Ag|AgCl, 1 M KCl|membrane|test solution|sat. KCl, AgCl|Ag) was measured alternately using a multichannel analog-to-digital converter. The pH of the test solution was also measured using a glass electrode (GE-10301/4, Econix-expert, Moscow, Russia).

**Figure 2:**
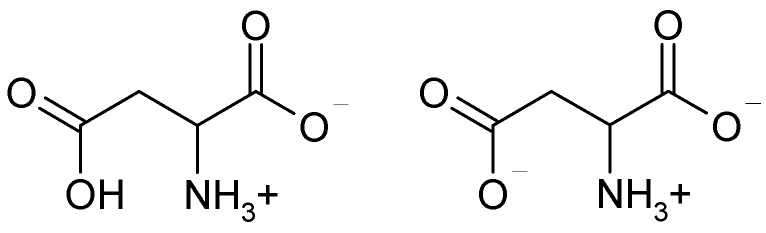
The structure of Asp^±^ and Asp^−^ ions.

The calibration of DP-sensors was performed in solutions containing aspartic acid and KOH with concentrations that varied from 1.0 × 10^−4^ to 1.0 × 10^−2^ M in the various proportions. Values of pH for solutions ranged from 3.99 to 8.20. The dissociation constants of functional groups for aspartic acid were equal to 1.88 (α-COOH), 3.65 (β-COOH), 9.60 (-NH_3_^+^). Therefore, the composition of solutions was of aspartic acid anions and zwitterions (Asp^−^, Asp^±^, Figure 2) and K^+^ ions.

The influence of the Asp^−^, Asp^±^, and K^+^ ions concentrations and the water dissociation products on the DP-sensors responses was taken into account in the calculation of the calibration equations coefficients by multivariate regression analysis:

Δϕ_D_ = *b*_0_ + *b*_1_*pK* + *b*_2_*pH* + *b*_3_*pAsp* (3)

where ∆ϕ_D_ is the DP-sensor response, mV; *pK* is the negative decimal logarithm of the K^+^-ion’s molar concentration; *pAsp* is the negative decimal logarithm of the asparaginate ions molar concentration; *b*_0_ is the free term of the calibration equation, mV; *b_i_* is the sensitivity coefficients of DP-sensor to the corresponding ions, mV/p*c*. The adequacy of the calibration equation was evaluated by the Fisher F-test to identify the possible systematic errors and to prove the correctness of its choice. The significance of the equation coefficients was evaluated by *t*-test.

**Figure 3:**
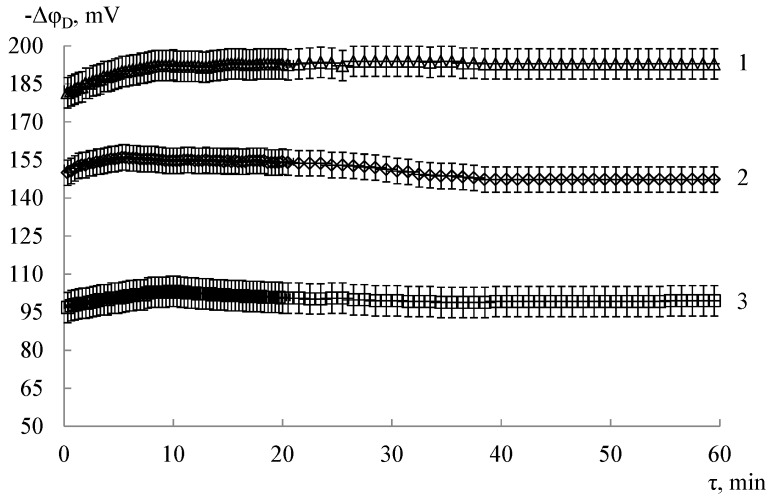
The dependence of response time of DP-sensor based on Nafion membrane in test solutions (*c*(Asp) = *c*(KOH), M): 1—1.0 × 10^−4^; 2—1.0 × 10^−3^; 3—1.0 × 10^−2^.

The response dispersion (s^2^, mV^2^) was determined to evaluate the reproducibility of DP-sensor response in the test solution. The stability of the DP-sensor’s response was performed based on the results of chronopotentiometric measurements over 1 h. The examples of the dependence of the DP-sensor response on time for the Nafion membrane are shown in Figure 3. A scatter of the response values during the measurement time was compared with the scatter of values at the experiment duplication to determine the sensory response time (τ_resp_, min). The change of the sensory response per unit time (mV/h) after the quasi-equilibrium establishment was evaluated to determine the response drift.

The system of calibration equations for a selected DP-sensors system (the number of equations is equal to the number of analytes) was solved to calculate the analyte concentrations in the model solutions and pharmaceutical. The experimental data for calculating concentrations were the response values of DP-sensors and pH values in the analysis object. The relative error (δ = (*c*_exp_ − *c*_theor_)/*c*_theor_, %) and the relative standard deviation (*s_r_* = *s*/*c*_exp_, %) were calculated to evaluate the accuracy and the reproducibility of analytes determination.

Panangin^®^ contains 45.2 mg/mL of potassium asparaginate hemihydrate, 40.0 mg/mL of magnesium asparaginate pentahydrate (active substances), and water for injection. The content of active substances in pharmaceutical corresponds to the Asp^−^, Asp^±^ and K^+^ ions concentration of 0.4727 and 0.2508 M, respectively. Pharmaceutical solutions for analysis were prepared by dilution with deionized water by 20, 200, and 2000 times.

## 3. Results and Discussion

### 3.1. Properties of Membranes

**Figure 4:**
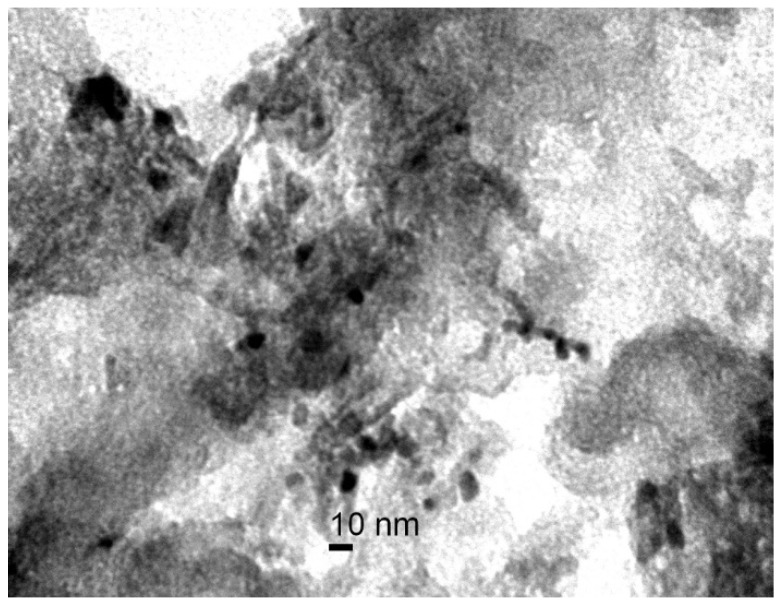
TEM micrograph of MF-4SC membranes + 3 wt.% SiO_2_ (5 mol.% R1) obtained via casting procedure [32].

Annealing of membranes shows that modification by casting procedure results in the formation of samples with dopant amounts close to the calculated (2.9 ± 0.1 wt.%). Modification of the previously prepared membrane (in situ procedure) allows to incorporate a smaller concentration of dopant (1.1 wt.%) because the formed membrane matrix can adsorb fewer amounts of bulk precursors during synthesis. TEM micrographs confirm the formation of nanoparticles with the size ranging from 4 to 10 nm (Figure 4). EDX analysis of hybrid membranes proves the presence of silica nanoparticles both in membranes obtained via casting and in situ procedures.

Since the cation exchange membranes are material for DP-sensors, then Asp^−^, Asp^±^ ions enter into the membranes through a non-exchange sorption. Therefore, the changes of the IEC, water uptake, and diffusion permeability of the membranes as a result of their modification and treatment were taken into account by the development of DP-sensors. The composition, treatment conditions, and the physico-chemical characteristics of the studied membranes are presented in Table 2 and Table 3.

The membrane modification by silica nanoparticles with the nitrogen-containing groups on the surface (SiO_2_(R)) leads to a similar change of the IEC and the water uptake depending on the membrane composition, both for materials obtained via an in situ procedure based on Nafion (Table 2) and for materials obtained by casting procedure based on MF-4SC (Table 3). The IEC for unmodified MF-4SC and Nafion membranes is about 0.9 mmol/g. The incorporation of dopant particles leads to a decrease in the membranes IEC. The decrease in the IEC is caused by the interaction of proton acceptor groups with functional sulfo groups and confirms the effectiveness of membrane modification. Moreover, the IEC is lower when the concentration of modifying groups on the SiO_2_ surface is higher. The IEC of the MF-4SC membranes obtained by casting procedure is 0.74 and 0.65 mmol/g for the SiO_2_(R1) nanoparticles and is 0.88 and 0.73 mmol/g for the SiO_2_(R2) nanoparticles with the concentrations of modifying groups of 5 and 10 mol.%, respectively [30]. IEC of hybrid membranes obtained via in situ procedure is reduced by five times compared with the unmodified Nafion. These changes are associated with the exclusion of the part of the membrane sulfo groups from the ion exchange due to the formation of bonds with dopant groups (NH_x_^+^…SO_3_^−^). In addition, sufficiently bulky modifying groups limit availability of the SiO_2_ surface and prevent its participation in the ion-exchange processes. This results in a significant decrease in IEC and, as a consequence, in a decrease in the membrane’s water uptake (Table 2 and Table 3). A greater decrease in IEC is observed for membranes with SiO_2_(R1) although the content of nitrogen atoms in the R2 group is more than twice compared with the R1 group. This is due to the fact that R2 groups are more bulky than R1, and a significant part of the nitrogen atoms is difficult to interact with membrane groups. Moreover, the water uptake of membranes containing the R2 group on the SiO_2_ surface is lower than of membranes containing the R1 group (Table 2 and Table 3) despite a higher IEC and a larger number of unbound sulfo groups. This may be due to the blocking effect of more bulky R2 groups. A decrease in the water uptake and the IEC is accompanied by a significant decrease in the anion transfer rate (diffusion permeability) for membranes obtained by in situ procedure (Table 2) as well as for membranes with a low modifying groups concentration (5 mol.%) obtained by casting procedure (Table 3). This is a consequence of a decrease in the charge carriers number, steric restrictions, and a decrease in the “electrically neutral” solution volume in the pores. Herewith, a diffusion permeability of samples with a bulky R2 group is lower than with an R1 group at the same molar content. This is in a good agreement with a water uptake change (Table 2 and Table 3). A diffusion permeability sharply increases with increasing the modifying groups concentration up to 10 mol.% in the samples obtained by casting procedure (Table 3). Its growth can be determined by an incomplete bonding of nitrogen-containing groups by hydrogen bonds, which acquire a positive charge during proton sorption and attract anions. Increase in the anion concentration results in the acceleration of their transfer rate in the membrane. The formation of bulky caverns arising during the membrane formation with the bulky dopant particles by casting procedure can be an alternative explanation [30]. Such process is unlikely for membranes obtained in situ.

An increase in the water uptake and the diffusion permeability of the unmodified membranes is achieved by their treatment in contact with water at t_ht_ = 120 °C (Table 2) [25]. In this case, the vapor pressure of water in sample environment is higher than the osmotic pressure inside it. This contributes to the stretching of membrane pores and channels, a sorption of additional water into them, and the increase in the volume of “electrically neutral” solution through which the anions diffuse. A treatment of the unmodified membranes at RH = 60% and t = 95 °C allows to achieve an irreversible decrease in their water uptake and diffusion permeability (Table 2). In this case, the pressure of water vapor outside the sample is lower than inside it, and part of water irreversibly exits from membrane. This is accompanied by a decrease in the pore size and, as a result, a decrease in channels connecting them, and a decrease in diffusion permeability. Deformation of unmodified membranes up to 80% in the dry state followed by the heating and hydration leads to the less decrease in water uptake than their treatment at RH = 60%, t = 95 °C (Table 2).

A significant decrease in the water uptake and the anion transfer rate is observed as a result of the treatment of membranes containing SiO_2_(R), both at RH = 60%, t = 95 °C, and at t_ht_ = 120 °C compared to the initial hybrid and unmodified membranes treated under the same conditions (Table 2). Probably, a presence of strong hydrogen bonds between the dopant groups and the membrane sulfo groups prevents an expansion of the pores and additional hydration under hydrothermal conditions. Herewith, a high treatment temperature t_ht_ = 120 °C leads to a polymer degradation. Since a larger number of sulfo groups are bound by R1 groups, a greater difference in membrane properties is observed between Nafion and Nafion + SiO_2_ (R1) samples treated under the same conditions.

### 3.2. Characteristics of DP-Sensors and Multisensory Systems

DP-sensors based on modified and treated MF-4SC and Nafion membranes are characterized by a low response time (τ_resp_ < 1 min), response dispersion (s^2^ = 1.6–60 mV^2^) and a response drift (1.7–10 mV/h) in solutions containing Asp^−^, Asp^±^, K^+^ ions in the analytes concentration range from 1.0 × 10^−4^ to 1.0 × 10^−2^ M at pH 3.99–8.20. The calibration characteristics of DP-sensors did not change over 1 year. This is because regeneration occurs completely after membrane conversion to the K^+^-form.

**Figure 5:**
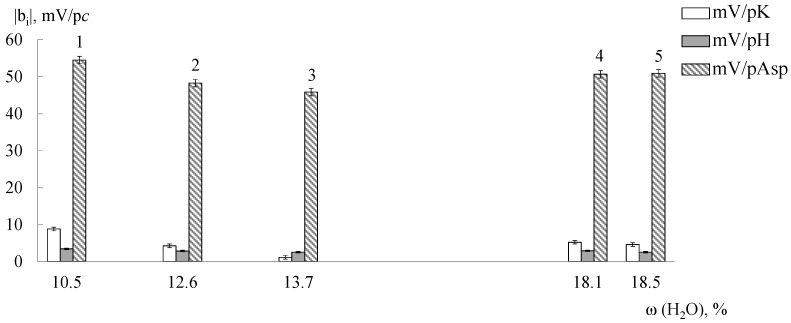
The dependence of DP-sensors’ sensitivity coefficients to ions in the Asp + KOH solutions (pH 3.99–8.20) on the water uptake (in the H^+^-form) for MF-4SC membranes + 3 wt.% SiO_2_(R) obtained via casting procedure: 1—10 mol.% R2; 2—5 mol.% R2; 3—10 mol.% R1; 4—initial sample; 5—5 mol.% R1.

**Figure 6:**
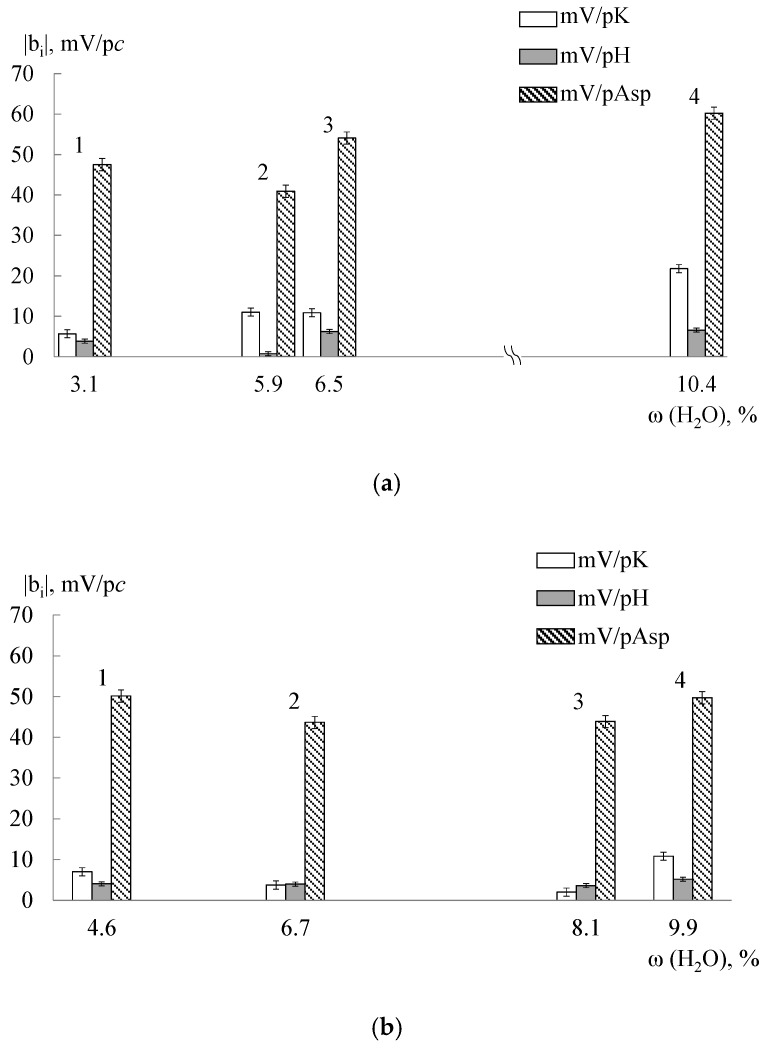
The dependence of DP-sensors’ sensitivity coefficients to ions in the Asp + KOH solutions (pH 3.99–8.20) on the water uptake (in the K^+^-form) of MF-4SC_(extrusion)_ (**a**) and Nafion_(extrusion)_ (**b**) membranes: 1—RH = 60%, t = 95 °C; 2—mechanical deformation of 80%, t = 80 °C; 3—initial sample; 4—t_ht_ = 120 °C.

**Figure 7:**
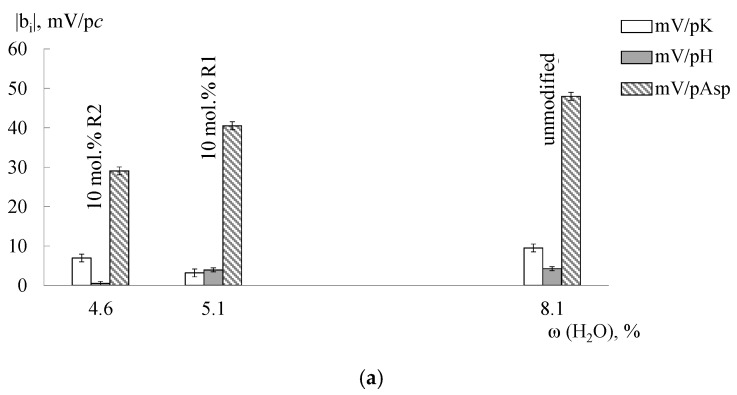

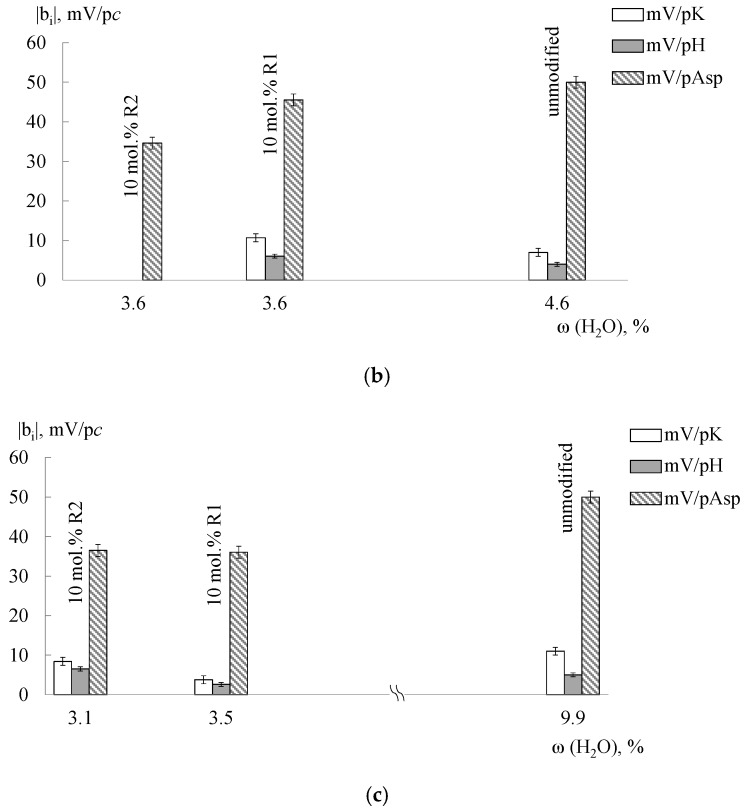
The dependence of DP-sensors’ sensitivity coefficients to ions in the Asp + KOH solutions (pH 3.99–8.20) on the water uptake (in the K^+^-form) of Nafion_(extrusion)_ + SiO_2_(R) membranes, without treatment (**a**), treated at the RH = 60%, t = 95 °C (**b**) and t_ht_ = 120 °C (**c**).

A sensitivity of DP-sensors based on both the initial, modified, and treated MF-4SC and Nafion membranes to Asp^−^, Asp^±^ ions significantly exceeds the sensitivity to K^+^ cations (Figure 5, Figure 6 and Figure 7). It should be noted that the pH of the solution inside the cation exchange membrane pores should be ~2 lower than the external one due to the Donnan exclusion of OH^−^ ions. The Asp^−^ anions almost completely transform into the zwitterionic form under these conditions and it will be repelled from the negatively charged pore walls by a lesser degree. Probably, electrostatic interactions and formation of hydrogen bonds (NH_3_^+^…SO_3_^−^ and COO^−^…K^+^…SO_3_^−^) between the analyte (NH_3_^+^, COO^−^) and the membrane groups exclude some K^+^ cations from the ion exchange. The efficiency of such interaction can be caused by the chelate effect due to the branched structure of Asp^±^ ions (Figure 2).

A tendency toward an increase in DP-sensors sensitivity to Asp^−^, Asp^±^ ions with an increase in the water uptake and the diffusion permeability of samples is observed in the most cases (Figure 5, Figure 6 and Figure 7), since non-exchange sorption is facilitated. A hydrothermal treatment of unmodified membranes makes a contribution to this (Figure 6) because an additional hydration and stretching of hydrophilic pores and channels lead to an increase in the number of bulky Asp^−^, Asp^±^ ions in the membrane and number of the sulfo-groups available for interaction with them. Additionally, a high sensitivity to Asp^−^, Asp^±^ ions of DP-sensors is achieved for the MF-4SC + 3 wt.% SiO_2_ (10 mol.% R2) sample (Figure 5). The diffusion permeability of this sample is higher and water uptake is lower as compared with the initial membrane (Table 3). This is in agreement with the assumption that this sample is characterized by larger pores and a higher number of nitrogen-containing groups available for interaction with the carboxyl groups of Asp^−^, Asp^±^. In addition, the interaction of asparaginate ions with the nitrogen-containing groups of the dopant changes the polarity of the double electric layer on the dopant surface. This leads to the expansion of pores due to the electrostatic dopant repulsion from the double electric layer at the membrane pore walls. This additionally facilitates non-exchange sorption.

A change of DP-sensors sensitivity to Asp^−^, Asp^±^ ions as a result of thermal treatment of Nafion + SiO_2_(R) hybrid membranes has features in comparison with the unmodified Nafion membrane (Figure 7). The incorporation of nitrogen-containing dopants leads to the “crosslinking” of the membranes and prevents an additional hydration during hydrothermal treatment. Thus, their pores are less accessible for Asp^−^, Asp^±^ ions. As a result, the maximum difference between the sensitivity of DP-sensors based on Nafion + SiO_2_(R) hybrid membranes to Asp^−^, Asp^±^ ions as compared with the unmodified Nafion membrane was observed for the samples treated in contact with water at 120 °C. However, it can be noted that the sensitivity of DP-sensors to Asp^−^, Asp^±^ ions increases for Nafion + SiO_2_(R) membranes treated at RH = 60%, t = 95 °C in comparison with untreated samples with the same composition. Moreover, a greater influence of the treatment conditions on the DP-sensors sensitivity was observed for membranes with R1 groups. The differences in the properties of these samples cause a decrease in the correlation between the responses of DP-sensors based on them. This allows to use them in the multisensory systems.

The pairs of DP-sensors with a high sensitivity to Asp^−^, Asp^±^ and K^+^ ions and the smallest correlation between the responses were chosen to organize multisensory systems. The following pairs of membrane compositions were selected: MF-4SC + 3 wt.% SiO_2_ (10 mol.% R1) and MF-4SC + 3 wt.% SiO_2_ (10 mol.% R2); Nafion and MF-4SC treated in contact with water at t_ht_ = 120 °C; Nafion treated at RH = 60%, t = 95 °C and Nafion + SiO_2_ (R2) treated at the same conditions. The characteristics of three multisensory systems determined in the analyte concentration ranged from 1.0 × 10^−4^ to 1.0 × 10^−2^ M and pH 3.99–8.20 are summarized in Table 4.

The highest reproducibility and accuracy of Asp^−^, Asp^±^ ion determination were obtained using an array based on MF-4SC membranes containing 3 wt. % of SiO_2_, modified by 10 mol% of R1 and R2 groups. The relative error (δ) and the relative standard deviation (s_r_) for determination of K^+^ cations were 0.2–16 and 7–21%, and for Asp^−^, Asp^±^ ions were 0.5–14 and 0.3–8%, respectively.

This multisensory system was used for determination of the active substances in Panangin^®^. The concentrations of Asp^−^, Asp^±^ ions in the test solutions were 2.364 × 10^−2^, 2.364 × 10^−3^, and 2.364 × 10^−4^ M, and for K^+^ ions were 1.254 × 10^−2^, 1.254 × 10^−3^, and 1.254 × 10^−4^ M, respectively. This corresponds to a drug dilution of 20, 200, and 2000 times, respectively, in accordance with its composition. The DP-sensor’s response, pH values, given and found values of Asp^−^, Asp^±^, K^+^ ions concentrations in drug solutions, as well as the errors of their determination are presented in Table 5. The calculation results of active substances concentration (mg/mL) in the pharmaceutical based on the found concentrations of Asp^−^, Asp^±^ and K^+^ ions are presented in Table 6. The relative error of the potassium asparaginate hemihydrate and the magnesium asparaginate pentahydrate determination was 2 and 4%, respectively.

## 4. Conclusions

Thus, the use of perfluorosulfonic acid cation exchange membranes containing silica nanoparticles with the proton-acceptor groups in a potentiometric multisensory system ensures the accuracy of asparaginate determination in the drug commensurate with the accuracy for known voltammetric sensors (Table 1) [1,2]. High stability (at least for 1 year), reagent-free analysis, relatively low pharmaceutical dilution, as well as the simplicity can be mentioned among the advantages of the proposed multisensory system for analysis of pharmaceuticals.

## Figures and Tables

**Table 1:** The characteristics of sensors for the determination of L-asparaginate ions in the model solutions, pharmaceutical and physiological environments [1,2,3,4,5].

Object	Method	Sensor Composition	*c*, mg/mL; *c*, M	*c*_min_, mg/mL; *c*_min_, M	Accuracy, %	Remarks	Ref.
Astymin Hepa	Differential pulse anode inversion voltammetry	Graphite electrode/TiO_2_ nanoparticles, MWCNTs/molecularly imprinted polymer membrane	(12.46–515.53) × 10^−6^; (0.09361–3.8733) × 10^−6^	1.73 × 10^−6^; 0.0130 × 10^−6^	96.1–101.3	A decrease in the selectivity upon reaching *i*_max_, a decrease in the response on 15% after 1 month of use, lifetime ~100 measurements	[1]
Blood serum			(9.98–524.25) × 10^−6^; (0.0750–3.9388) × 10^−6^	1.77 × 10^−6^; 0.0133 × 10^−6^	97.8–102.6		
Cerebro-spinal fluid *			(9.98–532.72) × 10^−6^; (0.0750–4.0024) × 10^−6^	1.79 × 10^−6^; 0.0134 × 10^−6^	98.0–101.1		
Astymin Hepa *		Graphite electrode/Au nanoparticles, MWCNTs/molecularly imprinted polymer membrane	(4.14–68.21) × 10^−6^; (0.0311–0.5125) × 10^−6^	1.16 × 10^−6^; 0.00872 × 10^−6^	99–102	A decrease in the response on 2.68%–2.71% after 3 weeks of use	[2]
Blood serum *			(4.30–69.38) × 10^−6^; (0.0323–0.5213) × 10^−6^	1.25 × 10^−6^; 0.00939 × 10^−6^	99–101		
Cerebro-spinal fluid *			(4.12–69.27) × 10^−6^; (0.0310–0.5204) × 10^−6^	1.17 × 10^−6^; 0.00879 × 10^−6^	99–102		
Model solution	Amperometry	Platinum electrode/malate-specific dehydrogenase, diaphorase	0.1331–1.331; 0.0010–0.0100	9.05; 0.068 × 10^−3^	-	The complexity of a stable enzyme layer formation, the influence of pH on sensitivity	[3]
Model solution	Cyclic voltammetry	Carbon paste electrode/Cu nanoparticles	(39.93–93.17) × 10^−3^; (0.300–0.700) × 10^−3^	3.993 × 10^−3^; 0.030 × 10^−3^	-	-	[4]
Model solution	Fluorimetry	Co (II)+2-(2-pyridyl) benzimidazole complex	1.331 × 10^−3^; 1.0 × 10^−5^	-	-	-	[5]

* Comparable characteristics are presented for D-asparaginate ions.

**Table 2:** The water uptake of membranes and the diffusion permeability of 0.1 M KCl solution into water through membranes in the K^+^-form (samples obtained by in situ).

Treatment Conditions	Composition	ω(H_2_O), %	P × 10^8^, cm^2^/s
Without treatment	MF-4SC	6.5	5.62
	Nafion	8.1	2.7
	Nafion + SiO_2_(R1)	5.1	0.21
	Nafion + SiO_2_(R2)	4.6	0.058
RH = 60%, t = 95 °C	MF-4SC	3.1	0.0067
	Nafion	4.6	0.12
	Nafion + SiO_2_(R1)	3.6	0.036
	Nafion + SiO_2_(R2)	3.6	0.052
t_ht_ = 120 °C	MF-4SC	10.4	16.2
	Nafion	9.9	32
	Nafion + SiO_2_(R1)	3.5	0.062
	Nafion + SiO_2_(R2)	3.1	0.034
Mechanical deformation of 80%, t = 80 °C	MF-4SC	5.9	- *
	Nafion	6.7	- *

* The studies were not carried out due to the inability to obtain samples with a size suitable for the experiment.

**Table 3:** The water uptake of membranes and the diffusion permeability of 0.1 M HCl solution into water through membranes in the H^+^-form (samples obtained by casting) [30].

Composition	ω(H_2_O), %	P × 10^8^, cm^2^/s
MF-4SC	18.1	53
MF-4SC + 3 wt.% SiO_2_ (5 mol.% R1)	18.5	47
MF-4SC + 3 wt.% SiO_2_ (10 mol.% R1)	13.7	270
MF-4SC + 3 wt.% SiO_2_ (5 mol.% R2)	12.6	22
MF-4SC + 3 wt.% SiO_2_ (10 mol.% R2)	10.5	130

**Table 4:** Characteristics of DP-sensors arrays for a determination of Asp^−^, Asp^±^, K^+^ ions in the analyte concentration range from 1.0 × 10^−4^ to 1.0 × 10^−2^ M at pH 3.99–8.20.

Array	I		II		III	
DP-sensor composition	Nafion RH = 60% t = 95 °C	Nafion 1.1–1.3 wt. % SiO_2_(R2) RH = 60 %, t = 95 °C	MF-4SC 3 wt.% SiO_2_ 10 mol.% R1	MF-4SC 3 wt.% SiO_2_ 10 mol.% R2	Nafion	MF-4SC t_ht_ = 120 °C
τ_resp_, min	<1					
Drift, mV/h	insignificant	insignificant	3 ± 1.5	5 ± 2	7 ± 5	insignificant
s^2^, mV^2^	50	30	1.6	9	60	50
*b*_1_, mV/pK	7.6 ± 0.8	insignificant	1.2 ± 0.6	8.8 ± 0.4	2.0 ± 1.2	21.8 ± 1.3
*b*_2_, mV/pH	4.6 ± 0.7	insignificant	2.56 ± 0.17	3.45 ± 0.12	3.6 ± 0.3	6.6 ± 0.4
*b*_3_, mV/pAsp	−53 ± 3	−35 ± 3	−45.8 ± 0.7	−54.5 ± 0.4	−43.9 ± 1.5	−60.2 ± 1.6
δ (K^+^), %	0.8–21		0.2–16		0.12–7	
δ (Asp^−^, Asp^±^), %	0.2–19		0.5–14		0.07–20	
s_r_ (K^+^), %	7–22		7–21		3–17	
s_r_ (Asp^−^, Asp^±^), %	3–20		0.3–8		0.4–15	

**Table 5:** The results of Asp^−^, Asp^±^ and K^+^ ions determination in Panangin^®^ solutions (*n* = 5, *p* = 0.95) using a multisensory system based on MF-4SC membranes containing 3 wt.% of SiO_2_ with 10 mol.% of R1 (DP-sensor 1) and 10 mol.% R2 (DP-sensor 2).

*c*_theor._, M		pH	−∆φ_D_, MB		*c*_exp_, M		δ, %		s_r_, %	
K^+^	Asp^−^, Asp^±^		DP-sensor 1	DP-sensor 2	K^+^	Asp^−^, Asp^±^	K^+^	Asp^−^, Asp^±^	K^+^	Asp^−^, Asp^±^
1.254 × 10^−4^	2.364 × 10^−4^	5.96 ± 0.06	182 ± 7	184 ± 3	(1.2 ± 0.2) × 10^−4^	(2.48 ± 0.18) × 10^−4^	1.5	5	17	7
1.254 × 10^−3^	2.364 × 10^−3^	6.21 ± 0.08	142 ± 4	142 ± 2	(1.21 ± 0.04) × 10^−3^	(2.01 ± 0.15) × 10^−3^	3	15	4	9
1.254 × 10^−2^	2.364 × 10^−2^	6.58 ± 0.04	92.1 ± 0.9	90.4 ± 1.5	(1.23 ± 0.11) × 10^−2^	(2.40 ± 0.05) × 10^−2^	2	1.5	11	3

**Table 6:** Calculations of the active substance’s concentration in Panangin^®^.

*c*_exp_, M (Solution)		*c*_exp_, mg/mL (Panangin^®^)	*c*_mean_, mg/mL (Panangin^®^)	δ, %	*c*_exp_, mg/mL (Panangin^®^)	*c*_mean_, mg/mL (Panangin^®^)	δ, %
K^+^	Asp^−^, Asp^±^	Potassium Asparaginate Hemihydrate			Magnesium Asparaginate Tetrahydrate		
(1.2 ± 0.2) × 10^−4^	(2.48 ± 0.18) × 10^−4^	44 ± 7	44	2	45 ± 14	38	4
(1.21 ± 0.04) × 10^−3^	(2.01 ± 0.15) × 10^−3^	43.8 ± 1.6			29 ± 7		
(1.23 ± 0.11) × 10^−2^	(2.40 ± 0.05) × 10^−2^	44 ± 4			42 ± 5		

## References

[1] Prasad B.B., Srivastava A., Tiwari M.P. (2013). Molecularly imprinted polymer-matrix nanocomposite for enantioselective electrochemical sensing of d-and l-aspartic acid. Mater. Sci. Eng. C.

[2] Prasad B.B., Jaiswal S., Singh K. (2017). Ultra-trace analysis of d-and l-aspartic acid applying one-by-one approach on a dual imprinted electrochemical sensor. Sens. Actuator B-Chem..

[3] Heli H., Hajjizadeh M., Jabbari A., Moosavi-Movahedi A.A. (2009). Fine steps of electrocatalytic oxidation and sensitive detection of some amino acids on copper nanoparticles. Anal. Biochem..

[4] Röhlen D.L., Pilas J., Schöning M.J., Selmer T. (2017). Development of an amperometric biosensor platform for the combined determination of L-malic, fumaric, and L-aspartic acid. Appl. Biochem. Biotechnol..

[5] Das S., Guha S., Banerjee A., Lohar S., Sahana A., Das D. (2011). 2-(2-Pyridyl) benzimidazole based Co (II) complex as an efficient fluorescent probe for trace level determination of aspartic and glutamic acid in aqueous solution: A displacement approach. Org. Biomol. Chem..

[6] Baldwin E.A., Bai J., Plotto A., Dea S. (2011). Electronic noses and tongues: Applications for the food and pharmaceutical industries. Sensors.

[7] Sidel’nikov A.V., Zil’berg R.A., Yarkaeva Y.A., Maistrenko V.N., Kraikin V.A. (2015). Voltammetric identification of antiarrhythmic medicines using principal component analysis. J. Anal. Chem..

[8] Kulapina E.G., Snesarev S.V., Makarova N.M., Pogorelova E.S. (2011). Potentiometric sensor arrays for the individual determination of penicillin class antibiotics using artificial neural networks. J. Anal. Chem..

[9] Wesoły M., Cetó X., Del Valle M., Ciosek P., Wróblewski W. (2016). Quantitative analysis of active pharmaceutical ingredients (APIs) using a potentiometric electronic tongue in a SIA flow system. Electroanalysis.

[10] Rahman M.M., Lopa N.S., Kim K., Lee J.J. (2015). Selective detection of L-tyrosine in the presence of ascorbic acid, dopamine, and uric acid at poly (thionine)-modified glassy carbon electrode. J. Electroanal. Chem..

[11] Ensafi A.A., Dadkhah-Tehrani S., Karimi-Maleh H. (2011). A voltammetric sensor for the simultaneous determination of L-cysteine and tryptophan using a p-aminophenol-multiwall carbon nanotube paste electrode. Anal. Sci..

[12] Liang R., Yin T., Qin W. (2015). A simple approach for fabricating solid-contact ion-selective electrodes using nanomaterials as transducers. Anal. Chim. Acta.

[13] Mercante L.A., Scagion V.P., Pavinatto A., Sanfelice R.C., Mattoso L.H., Correa D.S. (2015). Electronic tongue based on nanostructured hybrid films of gold nanoparticles and phthalocyanines for milk analysis. J. Nanomater..

[14] Facure M.H., Mercante L.A., Mattoso L.H., Correa D.S. (2017). Detection of trace levels of organophosphate pesticides using an electronic tongue based on graphene hybrid nanocomposites. Talanta.

[15] Teodoro K.B.R., Shimizu F.M., Scagion V.P., Correa D.S. (2019). Ternary nanocomposites based on cellulose nanowhiskers, silver nanoparticles and electrospun nanofibers: Use inan electronic tongue for heavy metal detection. Sens. Actuators B.

[16] Apel P.Y., Bobreshova O.V., Volkov A.V., Volkov V.V., Nikonenko V.V., Stenina I.A., Filippov A.N., Yampolskii Y.P., Yaroslavtsev A.B. (2019). Prospects of Membrane Science Development. Membr. Membr. Technol..

[17] Hwang D.W., Lee S., Seo M., Chung T.D. (2018). Recent advances in electrochemical non-enzymatic glucose sensors—A review. Anal. Chim. Acta.

[18] Bratov A., Abramova N., Ipatov A. (2010). Recent trends in potentiometric sensor arrays—A review. Anal. Chim. Acta.

[19] Kusoglu A., Weber A.C. (2017). New insights into perfluorinated sulfonic-acid ionomers. Chem. Rev..

[20] Bobreshova O.V., Parshina A.V., Polumestnaya K.A., Safronova E.Y., Yankina K.Y., Yaroslavtsev A.B. (2012). Perfluorinated sulfocation-exchange membranes modified with zirconia for sensors susceptible to organic anions in multiionic aqueous solutions. Mendeleev Commun..

[21] Parshina A.V., Denisova T.S., Safronova E.Y., Karavanova Y.A., Safronov D.V., Bobreshova O.V., Yaroslavtsev A.B. (2017). Determination of sulfur-containing anions in alkaline solutions using arrays of DP-sensors based on hybrid perfluorinated membranes with proton-donor dopants. J. Anal. Chem..

[22] Safronova E., Parshina A., Kolganova T., Bobreshova O., Pourcelly G., Yaroslavtsev A. (2018). Potentiometric sensors arrays based on perfluorinated membranes and silica nanoparticles with surface modified by proton-acceptor groups, for the determination of aspartic and glutamic amino acids anions and potassium cations. J. Electroanal. Chem..

[23] Berezina N.P., Timofeev S.V., Kononenko N.A. (2002). Effect of conditioning techniques of perfluorinated sulphocationic membranes on their hydrophylic and electrotransport properties. J. Membr. Sci..

[24] Alberti G., Narducci R. (2009). Evolution of permanent deformations (or memory) in Nafion 117 membranes with changes in temperature, relative humidity and time, and its importance in the development of medium temperature PEMFCs. Fuel Cells.

[25] Safronova E., Safronov D., Lysova A., Parshina A., Bobreshova O., Pourcelly G., Yaroslavtsev A. (2017). Sensitivity of potentiometric sensors based on Nafion^®^-type membranes and effect of the membranes mechanical, thermal, and hydrothermal treatments on the on their properties. Sens. Actuator B-Chem..

[26] Safronova E.Y., Stenina I.A., Yaroslavtsev A.B. (2017). The possibility of changing the transport properties of ion-exchange membranes by their treatment. Pet. Chem..

[27] Liu D., Hickner M.A., Case S.W., Lesko J.J. (2006). Relaxation of proton conductivity and stress in proton exchange membranes under strain. J. Eng. Mater. Technol..

[28] DeBonis D., Mayer M., Omosebi A., Besser R.S. (2016). Analysis of mechanism of Nafion^®^ conductivity change due to hot pressing treatment. Renew. Energy.

[29] Parshina A.V., Safronova E.Y., Titova T.S., Safronov D.V., Lysova A.A., Bobreshova O.V., Yaroslavtsev A.B. (2017). Potentiometric Cross-Sensitive Sensors Based on Perfluorinated Membranes Treated at Different Relative Humidity for Codetermination of Cations and Anions in Alkaline Solutions of Amino Acids. Russ. J. Electrochem..

[30] Safronova E.Y., Yaroslavtsev A.B. (2013). Relationship between properties of hybrid ion-exchange membranes and dopant nature. Solid State Ion..

[31] Safronova E.Y., Il’in A.B., Lysova A.A., Yaroslavtsev A.B. (2012). Effect of surface modification with carbon-containing groups on the size, properties, and morphology of silica particles. Inorg. Mater..

[32] Mikheev A.G., Safronova E.Y., Yurkov G.Y., Yaroslavtsev A.B. (2013). Hybrid materials based on MF-4SC perfluorinated sulfo cation-exchange membranes and silica with proton-acceptor properties. Mend. Comm..

